# Unraveling Human Brain Development and Evolution Using Organoid Models

**DOI:** 10.3389/fcell.2021.737429

**Published:** 2021-10-07

**Authors:** Sarah Fernandes, Davis Klein, Maria C. Marchetto

**Affiliations:** ^1^Laboratory of Genetics, The Salk Institute for Biological Studies, La Jolla, CA, United States; ^2^Department of Biological Sciences, University of California, San Diego, San Diego, CA, United States; ^3^Department of Chemistry and Biochemistry, San Diego State University, San Diego, CA, United States; ^4^Department of Anthropology, Center for Academic Research and Training in Anthropogeny (CARTA), University of California, San Diego, San Diego, CA, United States

**Keywords:** neurodevelopment, evolution, brain disorders, disease modeling, non-human primates, neocortex, transcriptomics, brain organoids

## Abstract

Brain organoids are proving to be physiologically relevant models for studying human brain development in terms of temporal transcriptional signature recapitulation, dynamic cytoarchitectural development, and functional electrophysiological maturation. Several studies have employed brain organoid technologies to elucidate human-specific processes of brain development, gene expression, and cellular maturation by comparing human-derived brain organoids to those of non-human primates (NHPs). Brain organoids have been established from a variety of NHP pluripotent stem cell (PSC) lines and many protocols are now available for generating brain organoids capable of reproducibly representing specific brain region identities. Innumerous combinations of brain region specific organoids derived from different human and NHP PSCs, with CRISPR-Cas9 gene editing techniques and strategies to promote advanced stages of maturation, will successfully establish complex brain model systems for the accurate representation and elucidation of human brain development. Identified human-specific processes of brain development are likely vulnerable to dysregulation and could result in the identification of therapeutic targets or disease prevention strategies. Here, we discuss the potential of brain organoids to successfully model human-specific processes of brain development and explore current strategies for pinpointing these differences.

## Introduction

There exists human (*Homo sapiens*)-specific molecular and cellular processes of brain development that are not shared even with our closest Hominidae relatives like chimpanzees (*Pan troglodytes*). While mammalian neurodevelopment mutually exhibits apicobasal organization of radial glia cells (RGs), timed cellular specification, and maturation of heterogeneous cell populations resulting in functional neural circuits (Campbell and Götz, 2002; Bakken et al., 2016), there are primate-specific characteristics of cortex development and maturation. These primate-specific features include delayed and extended periods of myelination, increased excessive synapse production, and postponed and protracted synaptic pruning (Petanjek et al., 2011; Miller et al., 2012). Furthermore, primates uniquely demonstrate expanded progenitor populations of the inner subventricular (iSVZ) and outer subventricular zones (oSVZ) which, together, comprise the subventricular zone (SVZ) (Giandomenico and Lancaster, 2017). It has been proposed that the advent of outer radial glial cells (oRGs) and the oSVZ led to the dramatic expansion of the cortex by giving rise to neurons of superficial layers and resulted in gyrification of the cortices of carnivores and primates, including humans (Stahl et al., 2013). Yet, recent findings in macaques support the hypothesis that, after embryonic day (E)92 and around the time of gyrification between E100 and E125, the oSVZ primarily functions as a source of astrocytes and oligodendrocytes rather than neurons (Rash et al., 2019). The complete coordination of neocortical expansion and gyrification is poorly understood as it is a complex process likely requiring the cumulative effect of several processes including neuronal growth, dendritic branching, epigenetic regulation, and the expansion of cortical neuropil (Hirabayashi and Gotoh, 2010; Rash et al., 2019).

Although primates commonly display features of brain development not observed in other mammals, it has been further suggested that approximately 9% of genes in humans have different developmental trajectories that could be contributing to delayed brain maturation when compared to rhesus monkeys (*Macaca mulatta*) (Bakken et al., 2016). Neoteny is thought to be central for the emergence of human-specific brain anatomy responsible for unique cognitive abilities by allowing for extended periods of neural remodeling, plasticity, and fine-tuning (Langer, 1996; Johnson, 2001). Pre- and postnatal transcriptomic analyses of human brain development have revealed neotenic gene expression patterns in humans when compared to macaques and chimpanzees; however, prenatal studies on neoteny are limited and increased sample distribution across age and developing brain regions are required to gain a more complete understanding of human neotenic brain development (Somel et al., 2009; Li et al., 2020). The timing of postnatal neotenic shifts in gene expression seem to correspond to the duration of gray-matter volume reduction which is associated with synapse elimination and remodeling (Somel et al., 2009). Synaptogenesis and the subsequent elimination of excessive synaptic spines are essential for optimal refinement of neuronal circuitry and cognitive skills while dysregulation of these processes have been linked to late-onset neuropsychiatric disorders (Petanjek et al., 2011). Studies suggest that both pre and postnatal human cortex development demonstrates regional differences and extended rates of synaptogenesis when compared to that of rhesus monkeys which exhibit uniform, simultaneous synaptogenesis (Rakic et al., 1986; Huttenlocher and Dabholkar, 1997). Humans are also born with reduced amounts of myelinated axons compared to chimpanzees and exhibit no adultlike neocortical myelination unlike chimpanzees (Miller et al., 2012). Additionally, dopaminergic (DAergic) axons, labeled by tyrosine hydroxylase (TH), show much higher innervation in layers V/VI of areas 9 and 32 of the human and chimpanzee cortex when compared to those of macaques (*Macaca*) (Raghanti et al., 2011). Altogether, human-specific aspects of neurodevelopment and maturation likely contribute to our differences in cellular and laminar organization, interconnectivity of various brain regions, hemispheric asymmetry, and overall encephalization resulting in species-specific cognitive abilities. Nonetheless, further research is needed to illuminate human-specific features of neurodevelopment and their correlation to distinctly human cognition (Preuss, 2011; Sakai et al., 2013; Table 1).

**TABLE 1:** Summary of some of the known and unknown aspects of human and NHP brain development and anatomy.

	**Differences in Brain Development and Anatomy**	**Unknowns of Brain Development and Anatomy**
Unique to humans	Neotenic gene expression patterns compared to NHPs	Complete map of neotenic gene expression patterns compared to NHPs, Systematic understanding of neoteny in humans compared to NHPs across age and developing brain regions, How neoteny gives rise to human-specific cognitive abilities
	Regional differences in rates of synaptogenesis in developing cortex (compared to rhesus monkeys)	How regional differences in rates of synaptogenesis differ in human when compared to NHP species other than rhesus monkeys
	Reduced amounts of myelinated axons at birth and no adultlike neocortical myelination at birth (compared to chimpanzees)	Developmental timing of axon myelination in human when compared to NHP species other than chimpanzees and across brain regions
	Higher innervation of dopaminergic axons (labeled by TH) in layers V/VI of areas 9 and 32 of the human cortex when compared to chimpanzees and macaques	While the involvement of DA in cognitive processes is well documented, how DAergic innervation corresponds to NHP differences in cognition and emotional processing is less understood
Unique to primates or other gyrencephalic species	Expanded regions and progenitor cell populations of the iSVZ and oSVZ	How expanded iSVZs and oSVZs contribute to expanded cortices and cognitive abilities
	oRGs comprising the oSVZ	The complete mechanisms by which oRGs contribute to cortical expansion and gyrification
	Denser, more extensive DAergic innervation with fibers in all cortical layers when compared to rodents	The specific mechanisms governing DAergic axon innervation in primates and humans during brain development

Uniquely human features of neocortical development and maturation are not only intriguing for their implications in human-specific cognitive abilities, but they are also vulnerable to dysregulation which could cause or contribute to distinctly human brain disorder pathophysiology. The human cerebral cortex is essential for both cognition and emotional processing and dysregulation of these processes of the cortex are associated with a wide range of brain disorders including schizophrenia (SZ), autism spectrum disorder (ASD), Parkinson’s disease (PD), and Alzheimer’s disease (AD) (Berman and Weinberger, 1991; Rubenstein, 2011; Xu et al., 2019). Much remains to be learned about the mechanisms governing cortical expansion and responses to pathogenesis between human and non-human primates (NHPs) (Otani et al., 2016). Understanding these differences could shed light on the underlying mechanisms responsible for human-specific brain disorders and lead to the identification of key targets for the development of effective therapies.

Subtle differences observed by comparing human neurodevelopment to that of our closest evolutionary relatives could reveal underlying mechanisms, including genomic or transcriptional differences, contributing to varied phenotypes (Pollen et al., 2019). Human-specific responses to pathogenesis might be elucidated in a similar manner; by comparing brain pathophysiology of humans to our non-human primate counterparts (Hof et al., 2004). Although rodent models have taught us much about basic mammalian brain development and disorders (Fernando and Robbins, 2011), comparing governing processes and responses to species more closely related to humans can reduce the number of variables allowing for the identification of specific mechanisms responsible for observed deviations. Studies analyzing induced pluripotent stem cells (iPSCs) derived from humans, chimpanzees, and bonobos (*Pan paniscus*) show large sets of differentially expressed genes between human and NHP iPSCs. Perhaps the most compelling differentially expressed genes are those related to increased long interspersed element-1 (LINE-1) mobility in chimpanzees and bonobos, which could have implications on the rates of genetic divergence among species, and alternative mechanisms of pluripotency maintenance in chimpanzees (Marchetto et al., 2013; Gallego Romero et al., 2015). Furthermore, when human and NHP iPSCs were differentiated to neurons, they displayed distinctive migratory patterns at the neural progenitor cell (NPC) stage followed by contrasting morphology and timing of maturation in neurons (Marchetto et al., 2019). Despite the ability of two-dimensional (2D) PSC-derived neural cultures to demonstrate basic organization and transcriptomic changes of early brain development (Yan et al., 2013), while retaining the genetic background of the somatic cells from which they are reprogrammed, they lack the ability to develop complex cytoarchitecture, recapitulate advanced spatiotemporal transcriptomics, and brain region interconnectivity (including migration and axon guidance) of ensuing primate brain development (Soldner and Jaenisch, 2019). Intricate cellular heterogeneity, complex architecture, and interconnectivity of neurodevelopment, in addition to pathogenic responses, could be observed by comparing human and NHP brain tissues; however, ethical concerns and the inaccessibility of pre- and postnatal primate brain tissues limits the feasibility of such studies.

While brain organoids might be a long way from forming or sharing thoughts with us, they could still teach us much about ourselves. Brain organoids are three-dimensional (3D), PSC-derived structures that display complex radial organization of expanding neuroepithelium following embedding in an extracellular matrix like Matrigel and can recapitulate some subsequent processes of neurodevelopment including neurogenesis, gliogenesis, synaptogenesis, heterogenous cytoarchitecture, cell and axon migration, myelination of axons, and spontaneously-active neuronal networks (Lancaster et al., 2013; Bagley et al., 2017; Birey et al., 2017; Quadrato et al., 2017; Xiang et al., 2017; Marton et al., 2019; Shaker et al., 2021). It is likely that all these features of neurodevelopment are governed by some degree of specifies-specific dynamics. Brain organoids can be generated from human and NHP PSCs and, since some pathways regulating neural induction and brain region specification are well conserved in primates, both unguided cerebral organoids and guided brain region specific organoids can be generated (Mora-Bermúdez et al., 2016; Field et al., 2019; Kanton et al., 2019). Additional protocols have been established for the derivation of brain region specific organoids from human PSCs (hPSCs), including dorsal forebrain, ventral forebrain, midbrain, thalamus, basal ganglia, cerebellum, and telencephalic organoids (Muguruma et al., 2015; Sakaguchi et al., 2015; Jo et al., 2016; Bagley et al., 2017; Birey et al., 2017; Watanabe et al., 2017; Xiang et al., 2017, 2019; Qian et al., 2018). With some modifications, these methods could prove to be successful in establishing brain region-specific organoids from a variety of NHP PSC lines allowing for the reproducible comparison of homogeneous, human-specific neurodevelopment and brain disorder pathophysiology in brain regions beyond the cortex.

**FIGURE 1:**
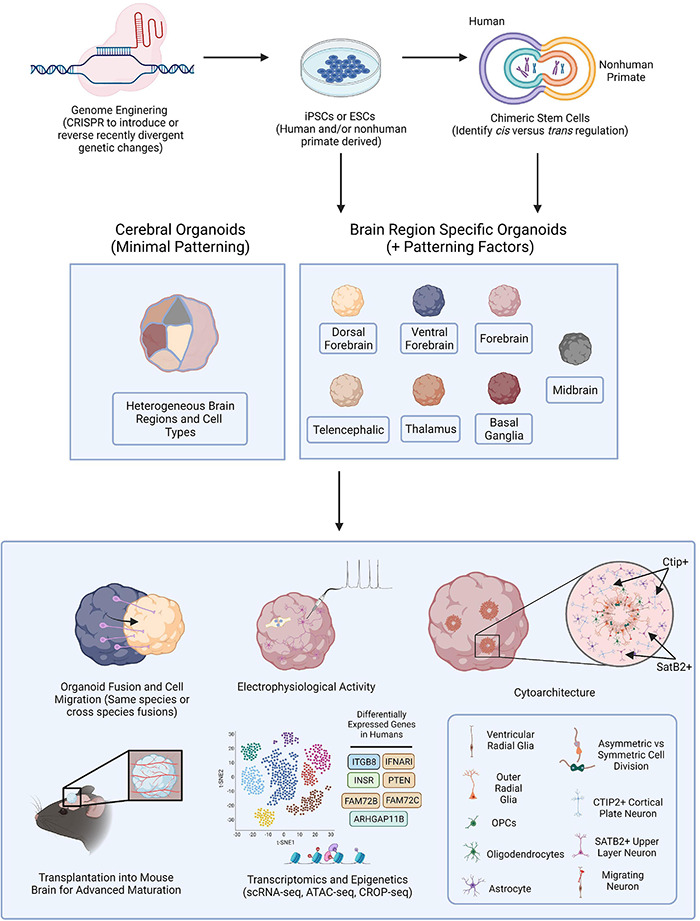
Brain Organoid Based Models to Identify Human-Specific Processes of Brain Development. Schematic outlining the possible methods for establishing brain organoids from PSCs and various means of analysis and downstream processes to compare resulting phenotypes. PSCs can be obtained from either human or NHPs and CRISPR-Cas9, or other gene editing technologies, can be employed to introduce genetic perturbations into the PSCs. Cerebral or brain region specific organoids can then be directly generated from PSCs or human and NHP PSCs can first be fused forming tetraploid hybrid PSCs for the identification of *cis* vs. *trans* regulatory elements and then be applied for the generation of brain organoids. Cerebral organoids or several different types of brain region specific organoids can be produced from a variety of human and NHP PSCs. Different combinations of organoids can then be fused to observe neuronal migratory patterns important for establishing healthy brain connectivity. Resulting organoids can be implanted into the mouse brain for subsequent vascularization allowing for oxygen and glucose penetration into the organoid and advanced cellular maturation. Human specific phenotypes, including complex, functional cytoarchitectural differences or differentially expressed genes can then be identified through different means of analysis including monitoring of electrophysiological activity using MEA or with high-throughput omic analyses like scRNA-seq or ATAC-seq.

In this review we discuss the application of human and NHP-derived brain organoids, and the implementation of genome editing technologies in these model systems, to investigate human brain evolution and pinpoint human-specific processes of neurodevelopment and maturation vulnerable to dysregulation (Figure 1). We further consider the application of brain organoid technologies to reveal distinctly human responses to pathogenesis. Our goal is to provide insight into methods for identifying uniquely human mechanisms driving brain disorder pathophysiology which could illuminate potential therapeutic targets.

## Brain Organoids Model Human Specific Brain Development and Evolution

Brain organoids are paving the way for *in vitro* access to elaborate, physiological relevant model systems of human-specific neurodevelopmental. Brain organoids have the advantage of retaining the species-specific genetic background of the PSCs from which they are derived while supporting the orchestration of dynamic processes of neurodevelopment as transcriptomic changes occur in a 3D space. PSCs have been established from a variety of NHP species including chimpanzee, bonobo, gorilla (*Gorilla gorilla*), orangutan (*Pongo abelii*), and rhesus monkey (Liu et al., 2008; Marchetto et al., 2013; Gallego Romero et al., 2015; Mostajo-Radji et al., 2020). Access to these cell lines sets the stage for creative brain organoid development and analysis representing several combinations of species, brain regions, and genomic and epigenomic modifications. Here, we discuss strategies to develop and analyze complex brain organoid model systems for the elucidation of distinctly human brain development and to provide insight into human brain evolution.

### Transcriptomics and Omics

The human brain is an immensely complex organ, with the prenatal cortex alone developing at a rate of approximately 3.86 million new neurons per hour including about 42.3 million synapses per minute, and requires over two decades to fully form into a structure containing approximately 84.6 billion communicating neurons (NeuN-positive cells) and 84.6 billion non-neuronal (NeuN-negative) cells (Azevedo et al., 2009; Silbereis et al., 2016). Although some inherent variation in human brain size and shape might occur, brain development is reproducibly accomplished through complex molecular and cellular processes that are governed by elegant regulation of a genetic blueprint (Silbereis et al., 2016; Reardon et al., 2018). These processes give rise to systematic transcriptional signatures that are expressed in consistent spatiotemporal patterns throughout neurodevelopment. How reliably brain organoids can recapitulate temporal cellular specification and maturation of brain development, indicated by changes in transcriptomics, is an ongoing conversation.

Bulk RNA-sequencing is useful for obtaining a sense of gene expression patterns and regulation across tissue or organoid samples; however, it is unable to discern transcriptomic trends at the single-cell level. Advances in single cell sequencing technology allow for transcriptome analysis with single cell resolution, providing detailed snapshots of transcriptomic changes and cellular heterogeneity during development or other dynamic tissue processes (Tang et al., 2009). Several platforms for single-cell RNA sequencing (scRNA-seq) are now available which can vary in sensitivity, accuracy, and precision (Ziegenhain et al., 2017). Beginning in 2015, various groups have employed scRNA-seq platforms to compare different brain organoid generation protocols among themselves and against human fetal brain tissues (Atamian et al., 2021). The earliest study used SMART-seq to contrast 226 single-cell transcriptomes from 12 and 13-week human neocortex against 333 single-cell transcriptomes from five cerebral organoids ranging from days 33 to 65. They found comparable gene expression profiles in over 80% of genes associated with neocortex disease or evolution between cerebral organoids and neocortical tissues (Camp et al., 2015). Following studies aimed to determine the reproducibility of the cellular composition of organoids by employing droplet-based single-cell mRNA sequencing (Drop-seq) to analyze about 67,000 cells from 19 optimized cerebral organoids isolated from four different bioreactors at 6 months. They reported a diversity of cell types in organoids closely related to those observed during *in vivo* brain development; however, although some cell types were reproducible generated, others appeared inconsistently which was partially attributed to bioreactor-based batch effects (Quadrato et al., 2017). More recently, researchers have used BD Rhapsody and 10X Genomics to analyze dorsal forebrain organoids, cortical spheroids, and human cortical organoids established *via* alternative directed differentiation protocols. While some groups noted an increase in apoptotic, glycolysis, and ER stress genes, they all cited the presence of reproducible cellular subtypes and aspects of development (Velasco et al., 2019; Yoon et al., 2019; Bhaduri et al., 2020; Tanaka et al., 2020).

scRNA-seq has led to a general consensus that brain organoids reliably recapitulate broad and some specific cell types present during neurodevelopment, transcriptional signatures of early to mid-fetal human cortex, and some degree of cellular maturation (Kelava and Lancaster, 2016; Sloan et al., 2017; Velasco et al., 2019). A recent study has reported the establishment of human cortical organoid protocols capable of achieving some epigenetic and transcriptional milestones up to postnatal days 250 and 300 (Gordon et al., 2021). These studies have laid the foundation for transcriptional comparisons of human and NHP derived organoids to elucidate evolutionary differences in gene expression patterns during brain development. Early reports describing the establishment of cerebral organoids from chimpanzee iPSCs turned to scRNA-seq to assist in characterizing the developed organoids. One group found populations of cells in chimpanzee organoids like those previously described in human cerebral organoids and with gene expression patterns correlated to those observed in human organoids and fetal cerebral cortex. The identified groups of cells in the chimpanzee cerebral organoids represented the cerebral cortex, hindbrain, ventral midbrain, and peripheral mesenchyme in addition to those in various stages of maturation (Camp et al., 2015; Mora-Bermúdez et al., 2016). Further analysis of the scRNA-seq transcriptome reads suggested that genes required for membrane structure, like integrin beta 8 (ITGB8), and intercellular signaling, including insulin receptor (INSR), were greatly upregulated in human organoid apical progenitor cells (APs) and neurons (Mora-Bermúdez et al., 2016).

More recent studies performed scaled-up scRNA-seq experiments with increasing numbers of brain organoid samples, cells, species, and timepoints. Using scRNA-seq (10X Genomics) to analyze human, chimpanzee, and macaque cerebral organoids at several timepoints from the PSC stage to 4 months of organoid development, researchers found that human neuronal development occurs at a protracted rate compared to chimpanzees and macaques and differentially expressed genes related to proliferation, neuronal migration, and neurite formation are enriched in human radial glial cells (RGs), intermediate progenitors (IPs), and neurons. Furthermore, accessible chromatin profiling demonstrated that differential chromatin accessibility corresponded to human-specific gene expression. Four of the 23 differentially expressed genes were specific to G2M phase progenitors of the telencephalon and included ARHGAP11B, FAM72B, FAM72C, and FAM72D (Kanton et al., 2019). An additional large-scale transcriptomic comparison of primary human and macaque telencephalon found 1,258 differentially expressed genes and, when these genes were contrasted with differentially expressed genes between human and chimpanzee telencephalon organoids using Fluidigm C1, 261 genes overlapped suggesting human-specific expression of these genes during cortical development. They identified upregulated gene co-expression modules in humans related to transcription during G1/S transition, neuronal apoptosis, and mTOR pathway genes (both activators, including INSR, ITGB8, and IFNAR1, and repressors like PTEN) implicated in stemness and long-term self-renewal (Pollen et al., 2019). Finally, cerebral cortex organoids were derived from human embryonic stem cells (ESCs), rhesus monkey ESCs, chimpanzee iPSCs, and orangutan iPSCs (Eiraku et al., 2008). Focusing on long non-coding RNAs (lncRNAs), associated with gene regulation, imprinting, and stem cell maintenance, the authors observed 920 human lncRNAs conserved in all species with transiently expressed lncRNAs in specific cell types as indicated through scRNA-seq (Field et al., 2019). Studies analyzing 33 brain regions from the postmortem tissue of humans, chimpanzees, bonobos, and macaques using bulk-RNA-sequencing and single nuclei RNA-sequencing (snRNA-seq) found that human-specific gene expression differences were not uniform across brain regions or cell types suggesting that even more cellular- and regionally-specified organoids could be advantageous in translating these evolutionary differences to function and brain development across species (Khrameeva et al., 2020).

scRNA-seq is powerful in characterizing known and unknown cell types, comparing tissue compositions, and understanding evolutionary differences at the cellular level; nevertheless, it requires the loss of spatial information which is necessary for elucidating the effects of transcriptomic differences on function and complex, physical cellular interactions as cells must be dissociated from their location in tissues. Through scRNA-sequencing analysis of four different lobes of the human neocortex, researchers found asynchronous cell development and a priority for regional maturation in the different areas emphasizing the importance of spatial resolution in conjugation with transcriptomic information (Fan et al., 2020). Furthermore, a large-scale study applying scRNA-seq to analyze 314 human microglia samples from 115 donors suggested that there are transcriptional differences among microglia from different brain regions and throughout aging (de Paiva Lopes et al., 2020). Although neurons in cortical and cerebral organoids do not seem to acquire spatial organization, progenitor and other cell types could and other brain organoid types might (Bhaduri et al., 2020; Libby et al., 2021). Neurons have been shown to display transcriptomic signatures of areal identity and considering these expression patterns could too be advantageous in analyzing transcriptomic information from organoids (Bhaduri et al., 2020). Spatial transcriptomic (ST) technologies are allowing researchers to merge transcriptomic and spatial information by employing glass slides printed with spatially barcoded oligo(dT) probes or bead arrays (high-definition ST) that permit mRNA hybridization to the probes after tissue slices are applied and enzymatically permeabilized (Vickovic et al., 2016, 2019; Larsson et al., 2021). Not only are ST technologies useful in relating transcriptomic changes to functional processes of brain development but they are also helpful in correlating transcriptomic changes that might occur during disease progression to cellular processes in response to pathogenesis. Utilizing the 5XFAD mouse model of AD, researchers implemented ST techniques to find that genes spatially associated with amyloid plaques had been previously identified as being functionally related to Amyloid-β (Choi et al., 2021). Implementing similar ST strategies to investigate AD and other brain disorders in organoid models derived from patients’ PSCs could reveal human-specific transcriptomic changes and their contribution to cell-specific responses to pathogenesis.

While evolutionary differences in gene expression have been analyzed primarily at the transcription level, understanding the influence of gene regulatory modalities at the epigenetic, protein, and microRNA level could illuminate processes governing human-specific neurodevelopmental phenotypes. Brain organoids have been demonstrated to cluster with fetal brain tissue and separately from adult brain tissue based on transcriptomes and histone marks (Amiri et al., 2018). They have also been shown to recapitulate transcriptomics and the epigenetic landscape of early to mid-fetal development (Luo et al., 2016). Researchers found that inhibition of G9a-mediated H3K9me2 modification in human cerebral organoids resulted in increased NPC proliferation causing organoid expansion, and G9a is likely regulated by the hominoid-specific protein TBC1D3 (Hou et al., 2021). These findings suggest an epigenetic regulatory mechanism for neocortical expansion and possibly ensuing gyrification. To understand how chromatin state varies in cells across tissues or organoids and influences gene expression in spatial patterns, researchers could implement high-spatial-resolution chromatin modification state profiling by sequencing (hsrChST-seq) which exploits DNA barcoding and Under Targets and Tagmentation (CUT&Tag) chemistry (Deng et al., 2021). Furthermore, applying multimodal omics analysis with combinations of techniques like single-cell ATAC-seq (scATAC-seq) with scRNA-seq, pooled CRISPR-Cas9 to introduce genetic perturbations followed by scRNA-seq or scATAC-seq, or simultaneous introduction of genetic perturbations with scRNA-seq (CROP-seq) could allow for the elucidation of regulatory relationship evolution during brain development (Datlinger et al., 2017; Efremova and Teichmann, 2020). Pooled CRISPR nuclease (CRISPRn) can target many genes introducing DNA breaks causing deletions or insertions that disrupt gene function while pooled CRISPR interference (CRISPRi) or CRISPR activation (CRISPRa) take advantage of nuclease dead Cas9 (dCas9) to interact with activator or repressor domains in DNA, modulating gene expression (Gilbert et al., 2013; Kampmann, 2017; Tian et al., 2019). Subsequent effects of such genetic perturbations during brain development can be compared between human and NHP species to elucidate human specific mechanisms of neurodevelopment. Additional regulatory elements of interest in human brain evolution include miRNA-mRNA interactions, in particular is the interaction between ORC4 (enriched in RGs) and miR-2115 (an miRNA specific to great apes), which is implicated in RG proliferation rates during human brain development (Nowakowski et al., 2018). Furthermore, researchers have developed a platform to divulge the influence of *cis* and *trans* acting regulation on gene expression. Human and chimpanzee iPSCs were fused so that the tetraploid genomic information contributed by each species shared the same nuclear space. Neural organoids were then established from the hybrid iPSCs and allele specific expression in hybrid organoids was compared to chimpanzee and human organoids to reveal *cis* regulatory divergences. Human somatostatin receptor 2 gene (SSTR2), responsible for neuronal calcium signaling regulation and associated with neuropsychiatric disorders, was found to be upregulated in these studies (Romero-Grimaldi and Moreno-López, 2008). Lastly, high-throughput mass spectrometry can be employed to obtain high resolution of the proteome of developed brain organoids and provide insight into the regulation of genes during neurodevelopment at the protein expression level (Bauernfeind et al., 2015; Mostajo-Radji et al., 2020).

### Cytoarchitecture

Much focus has been given to primate specific differences in neocortical development, yet there is less information on, but likely existing, primate and human specific differences in alternate brain regions that occur during development and maturation. For example, recent studies using computational neuroanatomy to reconstruct Neanderthal (*Homo neanderthalensis*) and early *Homo sapiens* brains found that Neanderthals, when averaged, had smaller cerebellar hemispheres than *Homo sapiens* which could have influences on cognition and social functions (Kochiyama et al., 2018). Human brains are not the largest on earth suggesting that evolutionary changes in brain structure, including the laminar organization of cells in the cortex, are particularly important in understanding species-specific cognition (Preuss, 2011; Sakai et al., 2013). Cellular organization and connectivity in the cortex and beyond vary among hominid species and are relevant in understanding differences in brain organization and function (Semendeferi and Damasio, 2000; Barger et al., 2007; Marchetto et al., 2019). Contributing to distinct brain cytoarchitecture and connectivity are unique cellular morphologies and subtypes like human and chimpanzee-specific varicose projection astrocytes, human-specific morphology of cortical pyramidal neurons, and primate-specific expression of TMEM14B in oRGs (Oberheim et al., 2009; Bianchi et al., 2013; Liu et al., 2017). Here, we discuss the current state and future applications of brain organoid-based models to recapitulate primate-specific brain regions, cytoarchitecture, and interconnectivity.

Unguided, cerebral organoid generation protocols promote the self-organization and expansion of developing neuroepithelium resulting in heterogeneous structures that represent several brain region identities. VZ-like structures of cerebral organoids are comprised of RGs with some oblique and vertical orientations which might be more indicative of human brain tissues than rodents. VZ-like zones also contain dividing cells at the apical surface. Additionally, TBR1^+^ cells show radial migration to developing preplate-like regions above an intermediate zone-like layer while TBR2^+^ cells indicate IP localization in SVZ-like structures (Lancaster et al., 2013). Expanded progenitor pools within these regions are thought to contribute to the diversified architecture of the developing cortex of primates and are valuable regions of interest in the identification of primate-specific processes of neocortical development (Giandomenico and Lancaster, 2017).

While cerebral organoid brain region heterogeneity could be applied to investigate brain region interconnectivity, cerebral organoids exhibit high amounts of variability among represented brain regions and more strictly guided protocols are often employed to achieve increased reproducibility and homogeneity. Forebrain and cortical organoids recapitulate some amount of timed NPC migration and neuronal subtype layering although they do not represent all six distinct layers of the primate cortex. Like cerebral organoids, forebrain organoids contain VZ-, SVZ-, preplate-, and CTIP2^+^ neuron-containing cortical plate (CP)-like structures; however, cortical organoid protocols have further demonstrated more distinct oSVZ-like regions and upper and deep cortical layers containing neurons and astrocytes reminiscent of third trimester human neocortical development (Lancaster et al., 2013; Kadoshima et al., 2014; Pasca et al., 2015; Qian et al., 2018, 2020). Neuronal migration events have been compared using human, chimpanzee, and macaque iPSC derived cortical organoids. Researchers observed the presence of abundant SATB2^+^ upper-layer neurons in macaque organoids at day 60 whereas human and chimpanzee organoids did not exhibit large populations of SATB2^+^ neurons in upper-layers until approximately day 80 of development corroborating their findings in 2D rosette systems (Otani et al., 2016). Furthermore, mitosis of APs at the ventricular surface of human iPSC-derived cerebral organoids were observed to experience a 40–60% increase in the time spent in metaphase when compared to chimpanzee and orangutan APs and it was determined that this phenomenon could be a feature of an earlier phase of cortical development. Additionally, S-phase was about 5 h longer in human APs than those of chimpanzees and orangutans (Mora-Bermúdez et al., 2016). Finally, immunohistochemical staining for the mTOR effector phosphorylated ribosomal protein S6 (pS6) in human, macaque, and chimpanzee-derived organoids revealed that oRGs of oSVZ-like regions of human organoids expressed higher amounts of pS6 indicating human-specific differences in mTOR signaling (Pollen et al., 2019). It is likely that observing primate-specific TMEM14B expression in oRGs of SVZ-like regions of human and NHP derived brain organoids could provide insight into cortical thickening and gyrification. It has been demonstrated that expressing TMEM14B in embryonic mouse NPCs induces thickening of the cortex and gyrification in the otherwise lissencephalic organism (Liu et al., 2017).

### Brain Region Specification

Brain organoids representative of additional brain regions and enriched with brain region specific cell types could illuminate evolutionary divergence in cellular morphology and dynamic processes of brain development. Researchers have applied what is known about *in vivo* brain development and *in vitro* 2D neural differentiation protocols to direct the differentiation of brain organoids *via* transcriptional regulation. Currently, brain region specific protocols are available for the development of midbrain, hypothalamus, hippocampus, cerebellum, and basal ganglionic organoids (Muguruma et al., 2015; Sakaguchi et al., 2015; Jo et al., 2016; Watanabe et al., 2017; Qian et al., 2018). Recently, caudalizing factors have been implemented during organoid generation to promote axial elongation similar to neural tube development. Remarkably, these hindbrain and neural tube organoids demonstrate regionalized gene expression and spatially distinct cellular organization showing promise that more definite cellular regionalization could be obtained in alternative brain region specific organoid models (Libby et al., 2021). Dorsal forebrain organoids enriched with excitatory glutamatergic pyramidal neurons and ventral forebrain organoids predominately containing inhibitory GABAergic cortical interneurons have also been established. By fusing dorsal and ventral forebrain organoids, researchers can observe the migration of ventral inhibitory neurons to targets in dorsal forebrain organoids (Bagley et al., 2017; Birey et al., 2017). This neuronal migratory event occurs during mid-to-late gestation and is essential for the integration and maturation of cortical circuits (Birey et al., 2017). Generating dorsal and ventral organoids from human and NHP PSCs and fusing both same species and cross species organoids could provide insight into evolutionary differences driving this significant process of brain development and maturation. Human-specific differences in pyramidal cell function and behavior, in addition to already observed morphological differences, might also be revealed by studying dorsal forebrain organoids enriched with excitatory pyramidal neurons (Marchetto et al., 2019). Furthermore, it could be possible to establish brain organoids enriched in DAergic, TH^+^ axons and observe potential human-specific patterns of DAergic axon innervation into fused cortical organoids (Raghanti et al., 2011). It is foreseeable that numerous combinations of human and NHP cerebral and brain region specific organoids could be developed and fused to elucidate the evolutionary differences responsible for brain interconnectivity and synchronization.

Human brain development is a prolonged process that continues well beyond prenatal events and into early adulthood (Silbereis et al., 2016). Implementing strategies that allow for long-term culture of complex brain organoid-based model systems are essential for obtaining a complete understanding of the processes responsible for the fabrication of the uniquely human brain. A reported lack of regional separation of neurons with areal identities and reduced neuronal maturation in brain organoids has been attributed to increases in oxidative and metabolic stress observed during *in vitro* culture (Bhaduri et al., 2020). Modifications to early cerebral organoid protocols, including seeding embryoid bodies with a reduced number of cells, incorporating a two-step neural induction phase, and supplementing final differentiation media with brain-derived neurotrophic factor (BDNF), allowed for progressive development of cerebral organoids over 9 months and advanced maturation of neurons with dendritic spines that formed spontaneously active neural networks (Quadrato et al., 2017). Other groups had found that the addition of recombinant leukemia inhibitory factor (LIF) during organoid generation increased the size of SVZ-like regions, allowed for increased separation of deep and upper layer neurons of the cortex, and maintained organoid structure to 22 weeks (Watanabe et al., 2017). More recently, researchers have accomplished brain organoid maintenance for up to 300 days demonstrating the ability to recapitulate later events of *in vivo* brain development like birth (Gordon et al., 2021). Since brain organoids lack a vascular network, overcoming hypoxic stress and reduced nutrient delivery into deeper layers of brain organoid structures has been an ongoing challenge. By culturing slices of forebrain organoids, researchers have achieved a reduction in the commonly occurring necrotic core of organoids, reduced apoptosis, more distinct separation of upper and lower cortical layers, and retention of VZ, oSVZ, and CP-like regions through days 100–150 (Qian et al., 2020). Additionally, developing organoids with a PSC derived perfusable vascular-like network, engineering organoids to develop an endogenous perfusable vascular-like network, or the transplantation of organoids into the mouse brain resulting in *in vivo* vascularization has greatly reduced apoptosis and cellular stress allowing for the development of organoids into the third trimester and advanced cellular maturation (Mansour et al., 2018; Pham et al., 2018; Cakir et al., 2019). Supporting the long-term culture of brain organoids could allow for the modeling of later processes of human-specific brain development, including overproduction of synapses followed by vigorous remodeling through synaptic pruning and delayed myelination of axons (Petanjek et al., 2011; Miller et al., 2012).

Gliogenesis, like neurogenesis, occurs both pre- and postnatally; however, the ability to model gliogenesis in brain organoid systems is limited due to reduced glial progenitor cell (GPC) populations when compared to primary neocortex tissue and the continued loss of GPCs as organoids develop (Stiles and Jernigan, 2010; Rusznák et al., 2016; Bhaduri et al., 2020). Preserving and enriching GPC populations in human brain organoids is necessary for the development of more representative models of human-specific brain development, as approximately half of adult brain volume consists of glial cells (Barres, 2008). Bioengineered neuronal organoids with induced neuro- and gliogenesis using FGF-2 for increased NPC proliferation, TGFb1 to promote gliogenesis, and DAPT for neuronal differentiation are enriched with astrocytes by day 60 and oligodendrocytes by day 150 (Zafeiriou et al., 2020). Astrocytes have also been reported in cortical spheroids, cerebral organoids, and forebrain organoids beginning between days 75 and 90; yet, the astrocytes typically do not represent the quantities found *in vivo* (Pasca et al., 2015; Quadrato et al., 2017; Renner et al., 2017; Sloan et al., 2017; Bhaduri et al., 2020). Observing astrocytes in glial-enriched organoids that exhibit advanced stages of maturation can reveal primate- and human-specific astrocyte, including chimp- and human-specific varicose projection astrocytes, influence on brain development and maturation (Oberheim et al., 2009).

Oligodendrocytes play a central role in healthy brain function by facilitating the electrical properties of neurons through the myelination of axons which occurs later and over a drawn out period of time in human development compared to chimpanzees (Barres, 2008; Miller et al., 2012). Oligodendrocytes are the last major neural cell type formed during human brain development; nonetheless, they have been established in brain organoids through both spontaneous formation and directed protocols that enrich for oligodendrocyte progenitor cells (OPCs) (Monzel et al., 2017; Renner et al., 2017; Matsui et al., 2018; Kim et al., 2019; Marton et al., 2019; Zafeiriou et al., 2020). Mature, functional oligodendrocytes have been identified in organoids *via* the expression of the mature oligodendrocyte marker, myelin basic protein (MBP), and the pinpointing of axons that are insulated by 2′,3′-cyclic nucleotide 3′-phosphodiesterase (CNP) positive oligodendrocyte myelin sheets (Monzel et al., 2017; Zafeiriou et al., 2020; Shaker et al., 2021).

However, the enrichment of GPCs might not result in the presence of microglia in organoids since microglia are derived from the mesoderm germ layer and likely migrate to the cerebrum sometime between the 4th and 24th week of development (Kierdorf et al., 2013; Menassa and Gomez-Nicola, 2018). Instead, the modification of organoid generation protocols to increase the number of mesodermal progenitors with already existing neuroectodermal progenitors has resulted in functional microglia with ramified morphology, a characteristic inflammatory response, and phagocytic ability including the capacity to perform synaptic pruning (Kierdorf et al., 2013; Menassa and Gomez-Nicola, 2018). Other options for incorporating microglia into brain organoids involve the coculture of primary microglia obtained from postmortem brain tissue or those derived from iPSCs. Implementing microglia derived from primary tissue or from the iPSCs of human and NHPs can help researchers to elucidate the behavior and influence of species-specific microglia interactions during development and could reveal species-specific microglia behaviors (Abud et al., 2017). For example, microglia from chimpanzees can be introduced into human brain organoids and the resulting effects on neuronal development can be compared to human organoids that contain human microglia to disentangle microglia influenced effects from cell autonomous effects. The significant roles that glial cells play in healthy brain development and maturation are just beginning to be appreciated and brain organoids might assist in further defining these elusive roles (Barres, 2008; Rusznák et al., 2016).

### Connectivity

Once complete brain organoid models are established, containing representative amounts of maturing cell types found in the developing brain under minimal cellular stresses, their electrophysiological activity can be monitored throughout development to observe oscillatory events during spontaneous network formation. While researchers have demonstrated spontaneous electrophysiological activity in cerebral organoids, it has only recently been suggested that human cortical organoids display oscillatory activity comparable to that recorded in preterm human electroencephalography (Quadrato et al., 2017; Trujillo et al., 2019). Previous works have applied multielectrode array (MEA) analysis to observe firing rates of human, chimpanzee, and bonobo iPSC-derived neurons at 2 and 6 weeks. They found increased firing rates of chimpanzee and bonobo derived neurons at 1.5 weeks when compared to human neurons, while human-derived neural cultures showed increased firing rates at 5 weeks (Marchetto et al., 2019). However, studies remain to be performed comparing human and NHP-derived brain organoid oscillatory activity to corroborate the finding of delayed functional maturation of human iPSC-derived 2D neurons compared to chimpanzee and bonobo neurons.

### Extinct Ancestral Modeling

Current work has brought us closer to our most recent non-living hominin relatives. Like discovering the DNA of an extinct species locked in amber, CRISPR-Cas9 has provided access to the past and opened possibilities for the interrogation of humans’ most recent divergences in brain evolution. Researchers identified neuro-oncological ventral antigen 1 (NOVA1) as a gene with differences between human and archaic hominin genomes that could play a role in human-specific neurodevelopment. Using CRISPR-Cas9, they replaced the human allele of NOVA1 in hiPSCs with an ancestral allele associated with Neanderthals and Denisovans. They then generated cortical organoids from edited hiPSCs with the archaic variant (NOVA1^*Ar/Ar*^) and from unedited isogenic control hiPSCs (NOVA1^Hu/Hu^). NOVA1^*Ar/Ar*^ organoids were smaller in diameter than NOVA1^Hu/Hu^ organoids during the proliferation and maturation stages and demonstrated increased surface rugosity. Additionally, NOVA1^Ar/Ar^ organoids had less VZ- and SVZ-like areas, increased apoptotic cells, and slower proliferation compared to NOVA1^Hu/Hu^ organoids. The researchers then performed RNA sequencing and found 277 genes differentially expressed between NOVA1^Hu/Hu^ organoids and NOVA1^Hu/Hu^ organoids with the top three differentially expressed genes being FEZF1, PAX6, and LHX5. Single-nucleus RNA-seq then revealed differences in cell-type proportions at one and 2 months of development and variations in gene splicing. Finally, NOVA1^*Ar/Ar*^ organoids had lower levels of pre- and postsynaptic proteins with less colocalized synaptic puncta likely resulting in reduced synchrony and increased variability based on observed firing rate and coefficient variation when evaluated by MEA (Trujillo et al., 2021).

Until very recently, Neanderthals and Denisovans (*Denisova hominins*) were thought to be our closest evolutionary relatives. The discovery of *Homo longi*, suspected to be an even closer extinct relative of humans than Neanderthals, could open the possibility for a new understanding of divergence in human brain evolution (Ji et al., 2021). Deviating genes might be identified from *Homo longi* that, 1 day, could be genome edited into human iPSCs for the development of different versions of ancestral organoids. Accurate cellular modeling of ancestral species will require recapitulation of ancestral genome mutations *via* introduction of multiple DNA edits in the same cell. Current CRISPR/Cas9 technology allows for multiple guide sequences to be encoded into a single CRISPR array to enable simultaneous editing of several sites within the primate genome (Cong et al., 2013). It remains to be seen if full recapitulation of ancestral mutations in a human neuron will change its physiology to a more ancient “Neanderthal-like” state.

## Brain Organoid Disease Modeling From an Evolutionary Perspective

**FIGURE 2:**
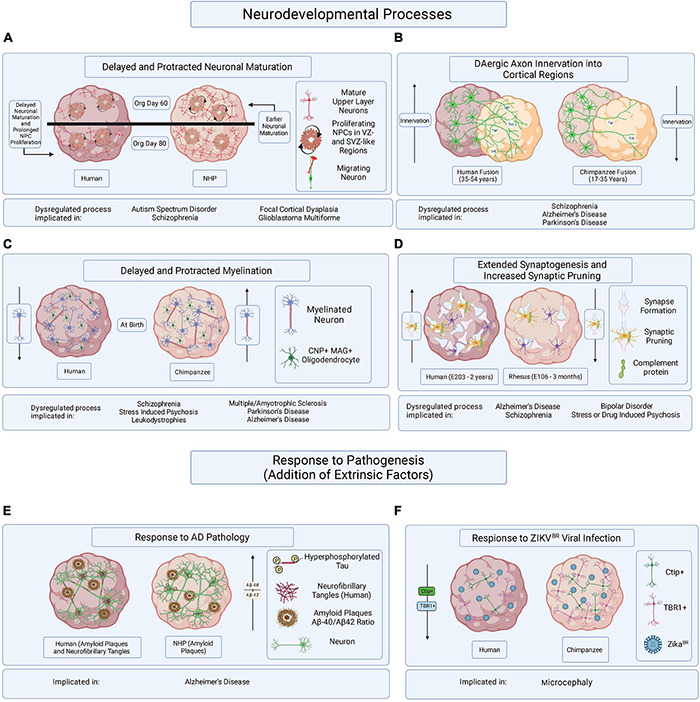
Hypothesized brain organoid-based models of human specific processes implicated in brain disease etiology and progression. Schematic illustrating multiple processes of human-specific brain development or responses to pathogenesis compared to those of NHPs and represented in brain organoids. **(A)** Delayed neuronal maturation and prolonged NPC proliferation have been observed in human brain organoids when compared to chimpanzee derived brain organoids. The dysregulation of NPC proliferation and neuronal maturation during brain developed has been linked to ASD, SZ, focal cortical dysplasia, and glioblastoma multiforme. Org is organoid. **(B)** It might be possible to observe differences in DAergic axon innervation between human and NHPs by fusing cortical organoids to organoids enriched in dopaminergic neurons. The disruption of DA regulation systems has been implicated in SZ, AD, and PD. Timepoints reflect observations of DAergic axon innervation in certain regions of the cortex comparing human and chimpanzee postmortem tissue samples. **(C)** Delayed and protracted myelination has been observed in human when compared to chimpanzee pre- and postnatal postmortem tissues and the disruption of axonal myelination is linked to SZ, stress induced psychosis, leukodystrophies, MS, PD, and AD. Axonal myelination might be compared in primate organoids containing CNP/MAG^+^ oligodendrocytes. **(D)** Observing periods of synaptogenesis followed by the pruning of excessive synapses in organoids containing microglia could reveal species-specific mechanisms of neuronal maturation when comparing primate organoids. Dysregulated synaptogenesis and synaptic pruning are implicated in AD, SZ, BD and stress and drug induced psychosis. E is embryonic day and timepoints correspond to observations in postmortem tissue. **(E)** It has been suggested that some aging NHPs develop amyloid-beta plaque deposits but only develop NFTs in rare cases. The overexpression of hyperphosphorylated tau, a precursor of NFT accumulation, in brain organoids derived from humans and NHPs could allow for the identification of species-specific responses to the disease burden. **(F)** Neurotrophic viral infection, like ZIKV^BR^ infection, can and has been compared among primate species by establishing human and NHP derived brain organoids, infecting them with neurotrophic viruses, and observing resulting differences in pathogenic responses or viral mechanisms of infection. The mentioned implicated brain disorders or processes are not all-encompassing.

Many of the previously discussed human-specific processes of brain development are vulnerable to dysregulation and could be at the root of human brain disorder pathophysiology or detrimental responses to pathogenesis (Table 2). Here we identify some potentially human-specific features of and responses to brain diseases and explore methods that might assist in elucidating therapeutic targets and protective strategies (Figure 2).

**TABLE 2:** Overview of primary literature employing human, NHP, and ancestral gene edited brain organoids to elucidate human-specific processes of brain development and implicated brain disorders.

	**Compared Species**	**Model**	**Findings**	**Implicated Brain Disorders**	**Citation**
Cortex development	• Chimpanzee	Brain organoids and 2D neural rosettes	Different rates of NPC proliferation; Differences in neurogenesis output; Cell autonomous regulation of neurogenesis	• Epilepsy	Otani et al., 2016
	• Macaque			• ASD	
	• Human			• HD	
				• TS	
				• FXS	
	• Chimpanzee	Cerebral organoids	Similar cytoarchitecture, cell type composition, and neurogenic gene expression programs; Lengthening of prometaphase-metaphase of AP mitosis; Differential AP gene expression associated with prolonged proliferative capacity; Less BPs in humans	• Microcephaly	Mora-Bermúdez et al., 2016
	• Human			• Lissencephaly	
				• Heteropias	
	• Chimpanzee	Cerebral (telencephalic) organoids	Protracted neuroepithelial differentiation in apes; Larger human organoids; Shorter cell cycles in humans; Differential gene expression of cell morphogenesis factors (ZEB2); Newly identified NPC transition morphotype state	• Mowat–Wilson syndrome (syndromic form of Hirschprung’s disease)	Benito-Kwiecinski et al., 2021
	• Gorilla				
	• Human				

Gene expression	• Chimpanzee	Cerebral organoids	Identified 216 differentially expressed genes in human vs. chimpanzees and macaque cortex (including regulators of PI3K/AKT/mTOR signaling); Increased activation of PI3K/AKT/mTOR pathway in human RGs dependent on two upregulated receptors in humans (INSR and ITGB8); Increase in almost all genes in the SMN1 and ARL17A loci in the developing human cortex of humans compared to chimpanzee and macaque	mTOR:	Pollen et al., 2019
				• ASD	
	• Macaque			• Focal cortical dysplasia	
	• Human			• Glioblastoma multiforme	
	• Chimpanzee	Cerebral oganoids	Human neuronal development happens at a slower pace; Identified 23 differentially expressed duplicated or rearranged genes in humans, four specific to G2M phase NPCs of the telencephalon (ARHGAP11B, FAM72B, FAM72C, and FAM72D); found 98 differentially expressed genes in RGs, IPs, and neurons and are associated with proliferation of RGs, neuron migration, and neurite formation; Differential accessibility in NPCs and neurons of humans and chimpanzees showed 7% increase and 9% decrease in accessibility in humans.	• ASD	Kanton et al., 2019
	• Macaque			• Microcephaly	
	• Human			• Lissencephaly	
				• Heteropias	
	• Chimpanzee	Hybrid human-chimpanzee, human, and chimpanzee PSC derived cortical spheroids	Thousands of genes with human vs. chimpanzee divergent *cis* regulation; Set of astrocyte related genes with evidence of selection; Upregulation of SSTR2 gene expression; Pharmacological induced species-specific calcium signaling	• Schizophrenia	Agoglia et al., 2021
	• Human			• Dementia in patients with AD Calcium signaling:	
				• AD	
				• PD	
				• HD	
				• ALS	
				• Spinocerebellar ataxias	
	• Neanderthal	CRISPR-Cas9 replaced human NOVA1 allele with ancestral NOVA1 allele in iPSCs, derived cortical organoids from archaic NOVA1 iPSCs and human iPSCs	Changes in alternative splicing of genes related to neurodevelopment, proliferation, and neural network formation; Archaic NOVA1 organoids had excitatory synaptic changes compared to human organoids; Smaller archaic NOVA1 organoids during the proliferation and maturation stages; Archaic NOVA1 organoids has less VZ- and SVZ-like areas, increased apoptotic cells, and slower proliferation; 277 differentially expressed genes between archaic NOVA1 organoids and human organoids (top three were FEZF1, PAX6, and LHX5)	• ASD	Trujillo et al., 2021
	• Human			• HD	
				• SZ	
				• Depression	
				• Cocaine addiction	

*List of implicated brain disorders is based on findings and is not exhaustive. ASD, autism spectrum disorder; HD, Huntington’s disease; SZ, schizophrenia; PD, Parkinson’s disease; AD, Alzheimer’s disease; TS, Timothy syndrome; FXS, fragile X syndrome.*

### Oligodendrocytes and Myelination

Contrasting human- and NHP-derived brain organoids that are optimized to model processes of human brain development vulnerable to dysregulation could allow researchers to elucidate the mechanisms governing those processes providing insight into brain disease etiology and potential therapeutic targets. For example, decreases in neocortical myelin development and myelin-related gene expression resulting in dysregulated CNP and myelin-associated protein (MAG) levels have been observed in patients with schizophrenia (SZ) (Hakak et al., 2001; Dracheva et al., 2006; McCullumsmith et al., 2007; Mitkus et al., 2008). Additionally, oligodendrocyte and astrocyte dysfunction has been associated with decreased myelination in multiple sclerosis, amyotrophic lateral sclerosis, PD, AD, Down syndrome (DS), and leukodystrophies (Salameh et al., 2015; Wasseff and Scherer, 2015; Domingues et al., 2016; McKenzie et al., 2017; Reiche et al., 2019; Shaker et al., 2021). It has been suggested that the extended period of myelination in humans, in addition to the metabolic burden of myelinating the large quantities of neurons in humans, increases our susceptibility to these brain diseases; however, the mechanisms responsible for the dysregulation of oligodendrocytes and their ability to maintain appropriate amounts of myelination for healthy brain function are unclear (Bove, 2018). Generating oligodendrocyte and astrocyte containing organoids capable of myelination from human and NHP PSCs could allow for the examination of species-specific molecular mechanisms governing myelination (Madhavan et al., 2018; Kim et al., 2019; Shaker et al., 2021). Investigating axon myelination through perturbations of the processes of myelination, like increasing metabolic stress or altering CNP and MAG gene expression, in both human and NHP organoids could reveal species-specific mechanisms of myelination homeostasis.

### Synaptogenesis

Additionally, synaptogenesis is a process of brain development with uniquely human features like high quantities of excessive synapse formation in the cerebral cortex followed by extended periods of synapse remodeling *via* pruning (Petanjek et al., 2011). The dysregulation of synaptic pruning by astrocyte and complement protein responsive microglia has been implicated in SZ, bipolar disorder (BD), and stress- or drug-induced psychosis (Feinberg, 1982; Stevens et al., 2007; Eroglu and Barres, 2010; Paolicelli et al., 2011; Petanjek et al., 2011; Sellgren et al., 2019). Incorporating microglia derived from human and NHP PSCs into brain organoids, establishing both same species and cross species microglia enriched organoid co-cultures, and inducing neuroinflammation *via* cytokine activation might reveal human-specific complement guided microglia responses during organoid development and synaptic pruning (Stevens et al., 2007; Severance et al., 2014; Abud et al., 2017). Microglia are also implicated in AD pathology and coordinate activities with astrocytes and other neural cells (Hong et al., 2016; Menassa and Gomez-Nicola, 2018). Transplanting brain organoids into the mouse brain has resulted in the colonization of the transplanted human brain organoids with ramified mouse microglia, and it might be feasible to inject PSC-derived human and NHP microglia into mouse brains containing AD patient iPSC-derived brain organoids to identify human-specific microglia, astrocyte, and blood brain barrier (BBB) interactions in an AD pathophysiological relevant environment (Abud et al., 2017; Mansour et al., 2018, 2021; Hasselmann et al., 2019; Park et al., 2021; Figure 1).

### Radial Glia and Neural Progenitor Cell Proliferation and Migration

Proliferating cells of the VZ-, SVZ-, and oSVZ-like regions of brain organoids could be of notable importance when investigating human-specific disease etiology. NPCs differentiated from the hiPSCs of SZ patients conserved a large amount of the gene signature of SZ hiPSC-derived neurons and demonstrated aberrant migration patterns compared to control NPCs which was observed in neurosphere outgrowth and microfluidic chamber migration assays (Brennand et al., 2015). Furthermore, brain organoids developed from the hiPSCs of patients with autism spectrum disorder (ASD) showed no difference in the ability of RGs to establish TBR1^+^ cortical neurons yet revealed increased thickness of CP-like regions and transcriptional dysregulation related to cortical neuron development. However, circumventing the NSC stage using direct iPSC to induced neuron conversion rescued abnormal neural fate specification and neurite branching (Schafer et al., 2019). Deviating migration patterns have been revealed in hiPSC-derived NPCs when compared to bonobo and chimpanzee NPCs in 2D cultures and evidence suggests that cortical progenitor cells play a key role in primate-specific cortical size through differences in the duration of cortical progenitor cell expansion (Otani et al., 2016; Marchetto et al., 2019). It has also been suggested that increased INSR and ITGB8 may be related to human-specific differences in mTOR signaling pathway activation in RGs, particularly oRGs, and mutations related to mTOR signaling have been linked to ASD, focal cortical dysplasia, and glioblastoma multiforme (Ceccarelli et al., 2016; Mora-Bermúdez et al., 2016; Pollen et al., 2019). These findings provide a platform for understanding the mechanisms regulating NPC proliferation, migration, and overall contributions to healthy human brain development through primate-derived brain organoid models.

## Discussion

Brain organoids provide unparalleled access to human-specific processes of neurodevelopment and these processes can be further highlighted by contrasting them with those of our closest NHP and extinct ancestral relatives. However, our access to ESCs and primary cells of some NHPs, including several species of lemurs, monkeys, and great apes, is limited due to declining species numbers (Gray et al., 2013; Estrada et al., 2017). Additionally, protocols to reprogram human somatic cells to PSCs, and to direct the differentiation of PSCs to neural and brain region specific lineages, could lack consistent translation to NHPs. Although early processes of transcriptional regulation during development are often well conserved across species, obtaining NHP brain organoids with increasing brain-region specification could pose a challenge and require species-specific optimization (Acampora et al., 1998; Godenschwege et al., 2006; Castells-Nobau et al., 2019). Cerebral and cortical brain organoids have been derived from chimpanzee, bonobo, rhesus monkey, and orangutan ESCs and iPSCs using protocols originally established for human PSC organoid derivation with minimal optimization (Mora-Bermúdez et al., 2016; Otani et al., 2016; Kanton et al., 2019; Pollen et al., 2019). Reconstructing and identifying diverging ancestral genes to introduce into human brain organoids for the modeling of evolutionary divergences in brain development is also challenging since ancestral DNA is often poorly preserved, fragmented, contaminated by other DNA molecules, and contains post-mortem mutations. Remarkably, recent progress in high-throughput sequencing technologies are transforming ancient DNA research by providing access to entire genome sequences from ancient DNA fragments (Orlando et al., 2015). Once brain organoids are developed from human and NHP PSCs, high-throughput scRNA-seq, scATAC-seq, CROP-seq, pooled CRISPR screens, and tandem mass spectrometry are revolutionizing our understanding of transcriptional, translational, and epigenetic regulations during human brain development and our ability to validate brain organoid model systems (Bakken et al., 2016; Amiri et al., 2018; Tian et al., 2019; Atamian et al., 2021). Eventually, the application of technologies like seqFISH might provide spatial-temporal resolution of dynamic transcriptional changes and cell lineage tracing in brain organoid models (Shah et al., 2018; Atamian et al., 2021).

Non-human primates brain organoid disease modeling could provide insight into human-specific brain disease etiology and pathogenesis. We have learned much from rodent models of human neurodevelopment and disease, including the idea that physiological differences between species could result in erroneous conclusions (Van Fernando and Robbins, 2011; Kariya and Ishikawa, 2019; Norman, 2019). However, modeling and comparing these physiological differences in species more closely related to us could help elucidate the human-specific processes implicated in brain disease progression. Human and NHP brain organoid-based models of AD could be exceptionally informative due to the unique susceptibility that humans seem to exhibit for developing AD, which could be related to genetic differences, cerebral structural differences, and increased lifespan (Walker and Jucker, 2017). Although aged NHPs, like chimpanzees, orangutans, rhesus monkeys, squirrel monkeys (*Samiri sciureus*), and gorillas, demonstrate diffuse amyloid plaques, neuritic amyloid plaques, and vascular amyloid, in addition to hyperphosphorylated tau expression in neurons, they most often do not present with tau positive neurofibrillary tangles (NFTs) or neuropil threads and very few NHPs have been reported with both amyloid beta pathology in combination with NFTs (Perez et al., 2013, 2016; Edler et al., 2017). The identification of cellular mechanisms providing NHPs with the capability of preventing or clearing NFT formation might be accomplished by challenging human- and NHP-derived brain organoids with over-expression of the shortest human brain tau isoform (T44) in neurons (Ishihara et al., 1999). This comparison could reveal potential therapeutic targets in AD. Additionally, it is unclear whether the response of astrocytes and microglia of AD patients is protective or detrimental to the neuroinflammatory pathogenesis of AD (Cai et al., 2017; Ahmad et al., 2019). Microglia and astrocyte activation are early responses to AD pathology that increase oxidative stress and the production of inflammatory cytokines, some of which exhibit neurotoxic effects (Heneka et al., 2014). By implementing previously discussed strategies to enrich brain organoids with astrocytes and microglia, one could investigate the complex astrocyte, microglia, and neuronal interactions in the context of AD related neuroinflammation. GFAP expression suggesting astrogliosis in chimpanzee brains does not appear to increase during aging as it does in humans suggesting lower levels of oxidative stress, and, while astrogliosis is observed in cortex layer I and the hippocampus of chimpanzees exhibiting AD pathology, it is not as widely spread throughout the cortex as has been observed in human postmortem AD tissues (Munger et al., 2020). It could be hypothesized that organisms with increasing brain complexity and resulting increased metabolic demand demonstrate greater susceptibility to oxidative stress during aging making them more inclined to develop age-related neurodegenerative diseases like AD, although more research is required to determine if there is a connection between brain complexity, metabolic demand, and oxidative stress handling. The lack of vascularization and hyper- and hypoxic conditions during *in vitro* brain organoid culture has been shown to cause increased metabolic, oxidative, and cellular stress compared to *in vivo* conditions resulting in increases in apoptosis and necrosis (Bhaduri et al., 2020). While this does present a challenge in establishing many physiological relevant brain organoid model systems, it remains to be tested if this effect might turn out to be an advantage by facilitating the exacerbation and early onset of disease phenotypes in brain organoids (Park et al., 2021).

In addition to the genetic and molecular basis of brain disease, the influence of external factors on brain development might also be compared in human- and NHP-derived brain organoids. For example, researchers infected human and chimpanzee cerebral organoids with the Brazilian Zika virus (ZIKV^BR^) and found a reduction in both TBR1^+^ and CTIP2^+^ cortical neurons, and a subsequent reduction in the size of the CP-like structure of human organoids following infection, but they did not observe these effects in chimpanzee organoids after ZIKV^BR^ infection suggesting human-specific neurotropism adaptations of ZIKV^BR^ (Cugola et al., 2016). These studies indicate that brain organoid models can be implemented in the study of neurotropic viral infections and subsequent influence on brain development. It is foreseeable that similar models might be applied to understand additional viruses suspected of reaching the brain and influencing brain development like Sars-cov2, HIV (through HIV-1 infected microglia), or cerebral malaria (Harbuzariu et al., 2019; Dos Reis et al., 2020; Ramani et al., 2020). Finally, toxicology screenings in human- and NHP-derived brain organoids could reveal compounds that have a species-specific influence on brain development and potentially identify the protective effect provided by the non-susceptible primate (Quadrato et al., 2016). Human and NHP-derived organoids can teach us much about ourselves and the world around us, from human-specific processes of brain development and disease to evolutionary differences and neurotropic viral mechanisms, possibly providing insight into the development of effective therapies and protective strategies against brain dysregulation.

## Author Contributions

SF wrote the manuscript. DK designed and generated the figures. MM conceived and supervised the work. All authors listed have made a substantial, direct and intellectual contribution to the work, and approved it for publication.

## Conflict of Interest

The authors declare that the research was conducted in the absence of any commercial or financial relationships that could be construed as a potential conflict of interest.

## Publisher’s Note

All claims expressed in this article are solely those of the authors and do not necessarily represent those of their affiliated organizations, or those of the publisher, the editors and the reviewers. Any product that may be evaluated in this article, or claim that may be made by its manufacturer, is not guaranteed or endorsed by the publisher.
